# Postoperative serum interleukin-6 levels correlate with survival in stage I-III colorectal cancer

**DOI:** 10.1186/s12876-023-02800-9

**Published:** 2023-05-16

**Authors:** Shouhan Feng, Zeshi Li, Mei Liu, Qianwen Ye, Tianhui Xue, Bing Yan

**Affiliations:** 1 Department of Oncology, Huzhou Traditional Chinese Medicine Hospital Affiliated to Zhejiang Chinese Medical University, Huzhou city of Zhejiang Province, 313000 P.R. China; 2 Department of Critical Care Medicine, Hainan Hospital of PLA General Hospital, Sanya city of Hainan province, 572000 P.R. China; 3 Department of Tumor Chemotherapy, Haikou People’s Hospital, Haikou city of Hainan province, 570208 P.R. China; 4 Department of Oncology, Hainan Hospital of PLA General Hospital, No. 80 of Jianglin Road, Haitang District of Sanya city, Hainan province, 572000 P.R. China

**Keywords:** Colorectal cancer, C-reactive protein, Procalcitonin, Interleukin-6, Survival

## Abstract

**Aims:**

The preoperative serum levels of inflammatory mediators, including C-reactive protein (CRP), procalcitonin (PCT) and interleukin-6 (IL-6), have been demonstrated to be correlated with patient outcomes in colorectal cancer (CRC); however, the prognostic role of these levels has been less well-studied in postoperative settings.

**Materials and methods:**

A total of 122 stage I-III CRC patients were retrospectively enrolled. Serum levels of CRP, PCT and IL-6 were measured after surgery, and their prognostic value was evaluated. Kaplan-Meier analysis was used to determine the differences in disease-free survival (DFS) and overall survival (OS) between patients with different levels of these mediators, and the Cox proportional hazards model was used to estimate the risk factors.

**Results:**

In contrast to CRP and PCT, only the level of IL-6 was significant in predicting DFS (P = 0.01) but not OS (P = 0.07). A total of 66.39% (81/122) of patients were assigned to the low IL-6 group and no significant differences were found in the collected clinicopathological parameters among the low or high IL-6 subgroups. The level of IL-6 was negatively correlated with postoperative (1 w) (R=-0.24, P = 0.02) absolute lymphocyte counts. Patients with low levels of IL-6 had better DFS (log rank = 6.10, P = 0.01) but not OS (log rank = 2.28, P = 0.13). Finally, the level of IL-6 was an independent risk factor for DFS (HR: 1.81, 95% CI: 1.03–3.15, P = 0.04).

**Conclusions:**

Compared to CRP and PCT, the level of IL-6 was observed to be the only significant factor in predicting the prognosis of stage I-III CRC patients after surgery, and a low level of IL-6 was associated with good DFS.

## Introduction

It has long been established that inflammation plays a critical role in cancer development, such as by facilitating the proliferation and survival of malignant cells and by promoting important biological processes such as angiogenesis and migration [1, 2]. Colorectal cancer (CRC), which exhibits increased mobility and mortality rates according to the latest statistics [3], is still a serious health problem worldwide. Previously, a substantial number of studies have indicated that inflammation-related mediators, such as C-reactive protein (CRP), procalcitonin (PCT) and interleukin-6 (IL-6), play an important role in the prediction of CRC prognosis.

Interestingly, most previous studies have explored the prognostic value of CRP, PCT and IL-6 levels individually for CRC in the preoperative setting [4, 5] and have yielded inconsistent results when considered together. For example, the study by Groblewska et al. indicated that CRP was the only independent risk factor for survival when compared to IL-6 [6]; in contrast, the study by Lee et al. suggested that IL-6 but not CRP, was an independent risk factor for disease-free survival (DFS) in stage III patients [7]. In addition, the study by Kwon et al. work suggested that both CRP and IL-6 were not independent risk factors for DFS or overall survival (OS) [8]. In recent years, a group of new CRC prognostic markers based on inflammatory cells have been identified; these examples include the neutrophil to lymphocyte ratio (NLR) and lymphocyte to monocyte ratio (LMR), and longitudinal measurements of these markers have suggested that they may be more meaningful when they were analyzed in postoperative settings [9, 10]. For CRP, PCT and IL-6, it is notable that many factors can cause changes in their levels during tumor removal, including surgical stress [11–13] and anesthesia [14, 15]. However, only a few studies have investigated their prognostic role concurrently in postoperative scenarios. For example, Hermunen et al. conducted a *post hoc* analysis for 147 patients (stages II-IV) in the phase III LIPSYT study and found that both postoperative CRP and IL-6 were significant for survival (CRP was a risk factor for DFS but not OS, and IL-6 showed opposite results) but with a low sensitivity (CRP: 20%, IL-6: 28% for DFS) [16]. Hua et al. performed a study with 306 stage II-III patients and found that IL-6 was the most promising prognostic marker in contrast with CRP [17]. Nonetheless, reports that concurrently explore the prognostic value of postoperative CRP, PCT and IL-6 levels in CRC are still lacking.

Based on this background, we aimed to concurrently examine the usefulness of postoperative CRP, PCT and IL-6 levels in determining the prognosis of stage I-III CRC.

## Methods

### Patient enrollment

Patients who underwent radical resection of colorectal adenocarcinoma from December 2012 to October 2020 at Hainan Hospital of PLA General Hospital were retrospectively enrolled. Patients who met any of the following criteria were not included: (1) accepted neoadjuvant therapies; (2) lacked any of the results of CRP, PCT and IL-6 levels within 7 days (d) (median 1 d; range:1–7 d) after surgery or routine blood tests in the subsequent 7 d and 2 weeks (w) to 3 months (m); (3) lacked any pathological TNM information or stage IV according to the 7th edition of the American Joint Committee on Cancer (AJCC) cancer staging manual; and (4) lost to follow-up. Other clinicopathological parameters were registered as described previously [18, 19]. The study was carried out in accordance with the principles stated in the Declaration of Helsinki and was approved by the ethics committee of Hainan Hospital of PLA General Hospital and the requirement for written informed consent was waived due to its retrospective nature.

### Routine blood tests and measurements of the target indicators

Routine laboratory tests and the method to measure blood cell fractions were performed as described previously [19, 20]. The CRP (reference: 0-0.5 mg/dl) was measured by using turbidimetric inhibition immunoassay with the CRPL3 kit (Roche Diagnostics GmbH, Mannheim, Germany) according to the manufacturer’s manual with the automatic analysis system (Cobas e 501, Roche, Switzerland). In addition, PCT (reference: <0.05 ng/ml) and IL-6 (reference: 0–7 pg/ml) were measured via chemiluminescence with the Elecsys BRAHMS PCT kit and Elecsys IL-6 kit (Roche Diagnostics GmbH, Mannheim, Germany) according to the manufacturer’s manual with the automatic analysis system (Cobas e 601 and Cobas e 801, Roche, Switzerland, respectively). The NLR and LMR were calculated as described previously [21].

### Endpoints

The follow-up was started immediately after resection according to the procedure described previously [19]. DFS and OS were set as the endpoints for the current study, and DFS was estimated from the time of operation to the time of any recurrence or metastasis or death from any cause; in addition, OS was estimated from the time of operation to the time of death from any cause. The latest follow-up point ended in December 2021.

### Statistical analysis

The significance of CRP, PCT and IL-6 in predicting survival was determined by using the receiver operating characteristic curve (ROC), and patients were then divided into low or high subgroups based on the optimal discriminator points when it was statistically significant. Differences in the collected clinicopathological parameters among these subgroups were analyzed via χ2-test or Student’s t test. Correlations of the levels of markers with the ALCs were estimated by using the Pearson or Spearman (when any of the parameters did not meet the Gaussian distribution) correlation coefficients. Moreover, differences in DFS and OS among these subgroups were calculated via Kaplan-Meier analysis. Risk factors for DFS and OS were determined by using the Cox proportional hazards model (with the iterative forward LR method). The statistical analyses were performed by using SPSS 20.0 (SPSS Inc., Chicago, IL, USA) and GraphPad Prism 5 (GraphPad Software Inc., San Diego, CA, USA). Two-sided P < 0.05 was regarded to be statistically significant.

## Results

### General features of the study cohort and the prognostic significance of CRP, PCT and IL-6

**Fig. 1:**
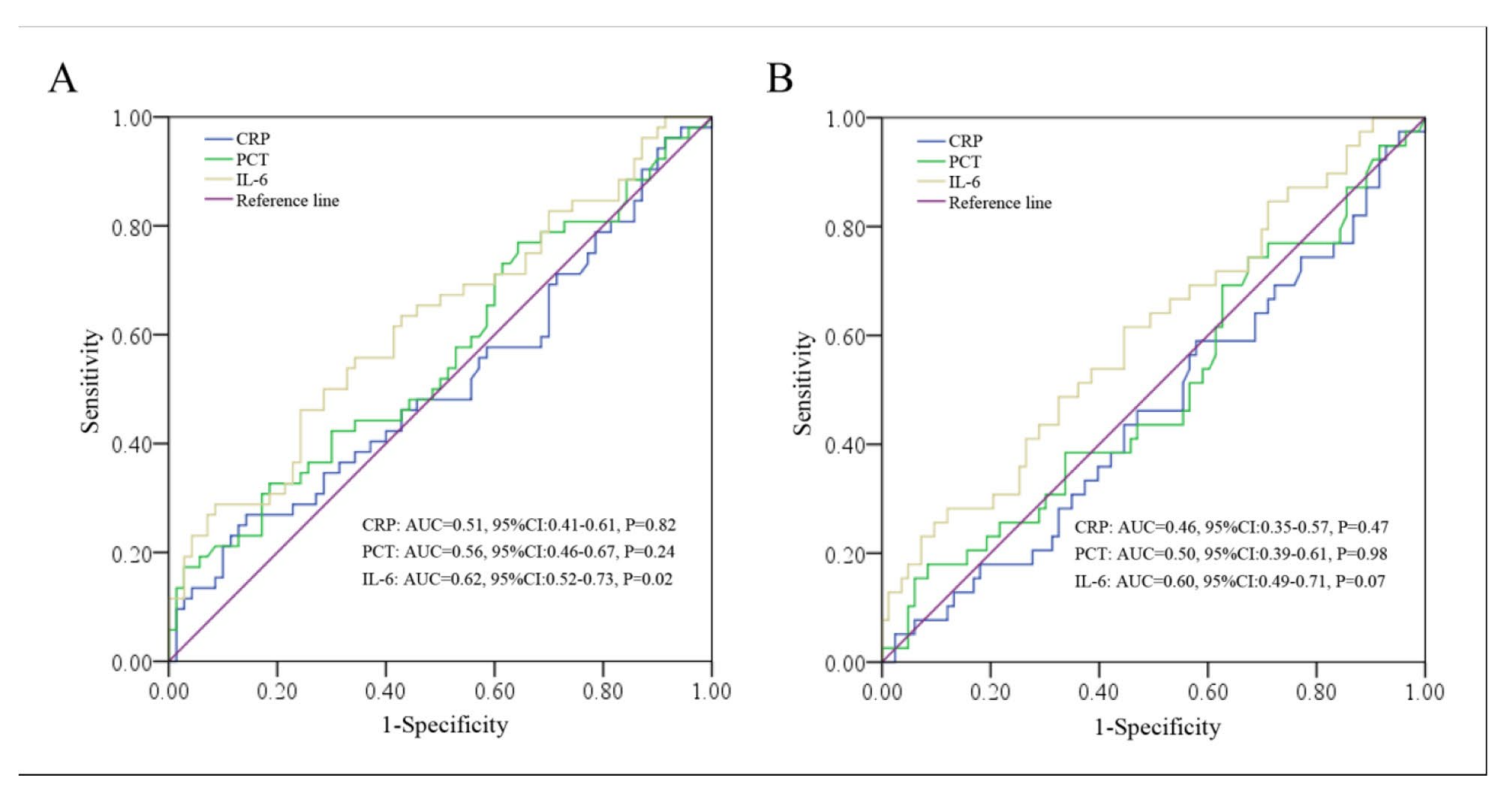
ROC results of CRP, PCT and IL-6 in predicting DFS **(A)** and OS **(B)**

A total of 122 patients were enrolled in the study. There were 44 female and 78 male patients, with a median age of 64 years (y) (range: 26–86 y) and a median follow-up of 44 m (1-108 m). At the end of the follow-up, 52 events were observed with 39 deaths (1 stage I patient, 12 stage II patients and 26 stage III patients). As shown in Fig. 1, in contrast to CRP and PCT, only the level of IL-6 was found to be significant in predicting DFS (area under the curve [AUC] = 0.62, 95% CI: 0.52–0.73, P = 0.02) but not OS (AUC = 0.60, 95% CI: 0.49–0.71, P = 0.07). The optimal discriminator point for IL-6 in predicting DFS was 70.27 pg/ml, and 66.39% (81/122) of patients were then assigned to the low IL-6 group (< 70.27 pg/ml) and 33.61% (41/122) of patients were assigned to the high IL-6 group (≥ 70.27 pg/ml). In addition, no differences were found for the collected clinicopathological parameters among the low or high IL-6 subgroups (Table 1).

**Table 1:** Comparison of the clinicopathological features among low or high level IL-6 subgroups

		IL-6 level		
	**No.**	**Low**	**High**	P
**Age (y)**				0.05
< 60	48	37	11	
≥ 60	74	44	30	
**Gender**				1.00
Male	78	52	26	
Female	44	29	15	
**Type of operation**				0.16
Laparotomy	26	14	12	
Laparoscopy	96	67	29	
**Tumor location**				0.52
Right	32	23	9	
Left	90	58	32	
**History of smoking**				0.10
Current + past	39	30	9	
Never	83	51	32	
**History of drinking**				0.43
Current + past	46	33	13	
Never	76	48	28	
**Histological grade**				0.42
Well + moderate	105	68	37	
Poor	17	13	4	
**Mucinous elements**				0.63
Without	101	66	35	
With	21	15	6	
**T stages**				0.43
T_1_ + T_2_	19	11	8	
T_3_ + T_4_	103	70	33	
**N stages**				0.34
N_0_	67	47	20	
N_1+_N_2_	55	34	21	
**TNM stages**				0.34
I + II	67	48	20	
III	55	34	21	
**BMI (kg/m** ^**2**^ **)**	122	23.34 ± 3.09	22.62 ± 3.29	0.23

No: number; BMI: body mass index

### Correlation of IL-6 with ALCs

**Fig. 2:**
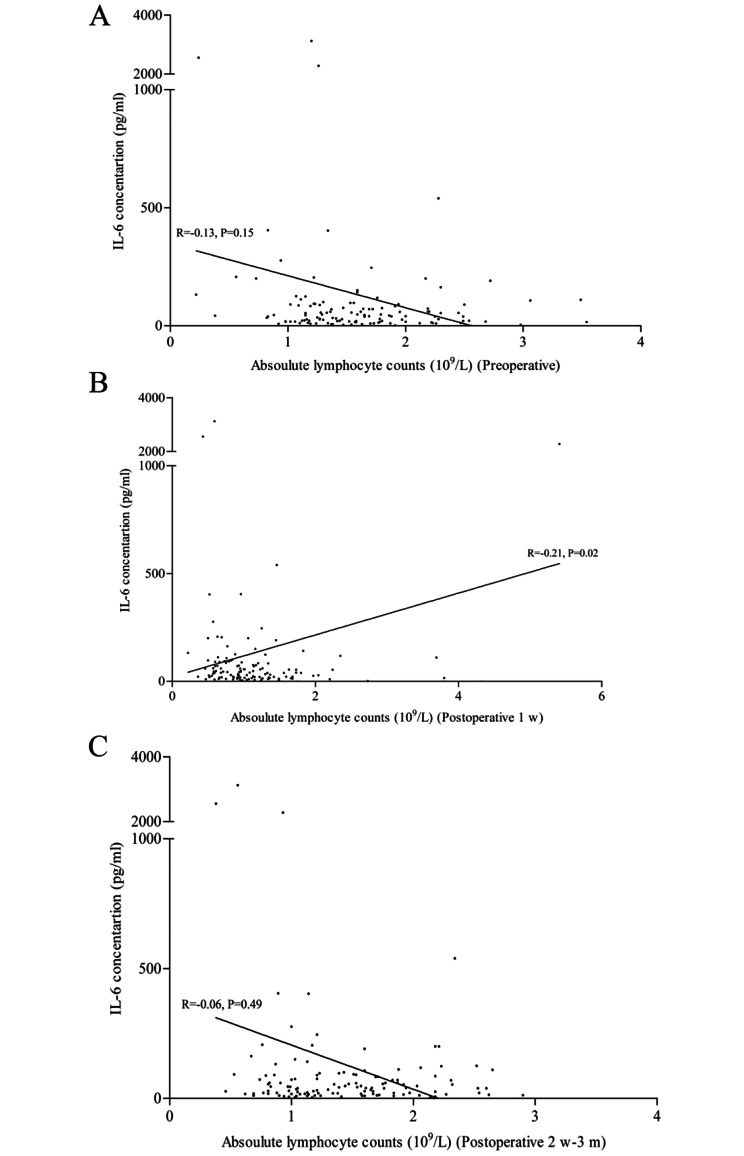
Correlation of postoperative IL-6 level with preoperative **(A)**, postoperative (1 w) **(B)**, (2 w-3 m) **(C)** absolute lymphocyte counts

By using the Spearman correlation analysis (Fig. 2), no significant correlations were observed for the level of IL-6 with the ALCs both preoperatively (R=-0.13, P = 0.15) and postoperatively at 2 w-3 m (R=-0.06, P = 0.49); however, a significant correlation was found for the level of IL-6 with the ALCs postoperatively (1 w) (R=-0.21, P = 0.02). Furthermore, significant positive correlations were observed between the levels of IL-6 and CRP (R = 0.52, P < 0.01) and PCT (R = 0.47, P < 0.01).

### Survival differences among low or high IL-6 subgroups

**Fig. 3:**
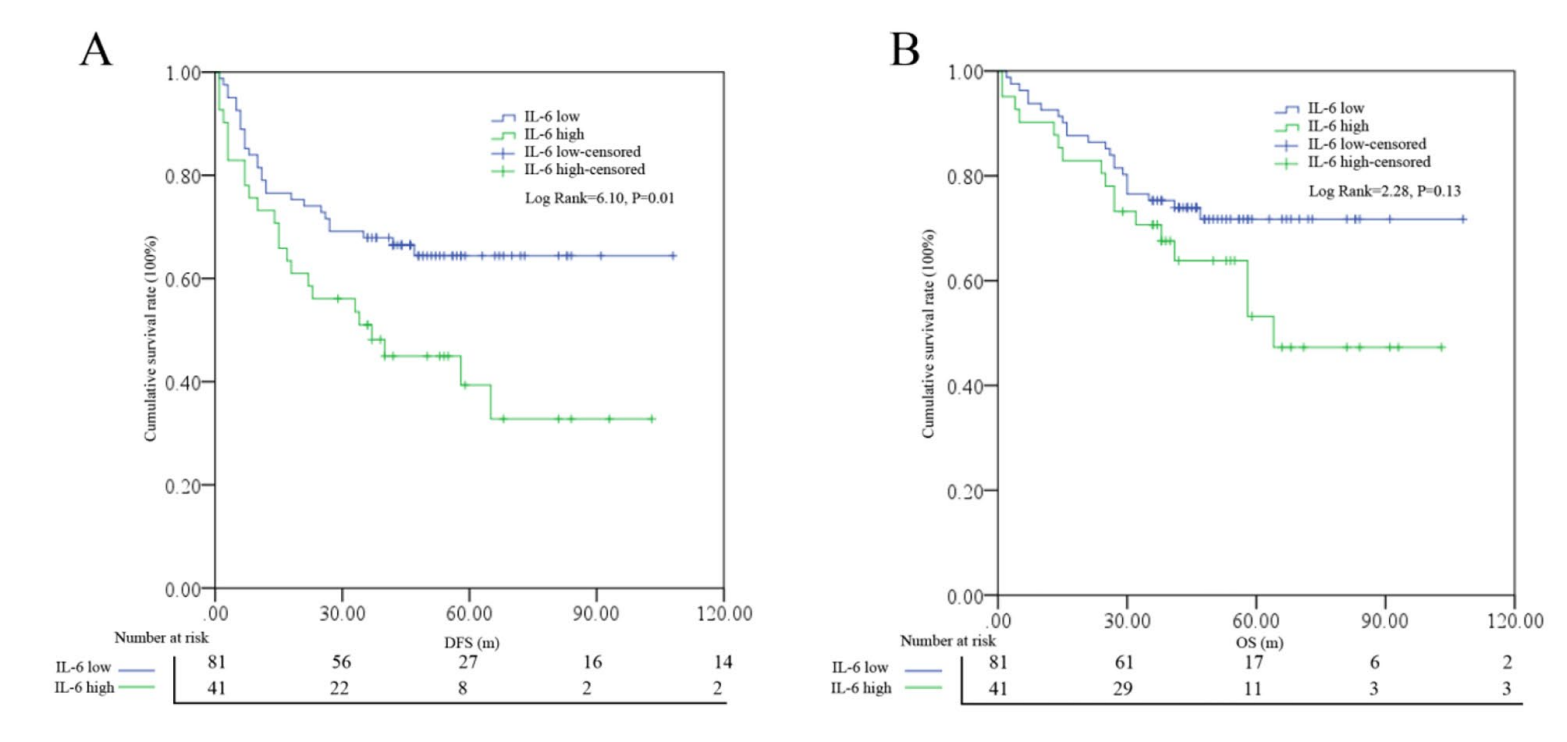
Survival differences for DFS **(A)** and OS **(B)** among low- or high-level IL-6 subgroups

According to the Kaplan-Meier analysis (Fig. 3), patients in the low level IL-6 group had significantly superior DFS (log rank = 6.10, P = 0.01) compared with the high level group; however, no such difference was found in OS (log rank = 2.28, P = 0.13).

### Risk factors determined by univariate and multivariate tests

By using the univariate tests, parameters including type of operation, N stage and TNM stage were demonstrated to be risk factors for both DFS and OS, whereas gender, history of drinking and IL-6 level were found to be additional risk factors for DFS (Table 2). Furthermore, when these factors were entered into the multivariate tests, the IL-6 level was found to be an independent risk factor for DFS (HR = 2.24, 95% CI: 1.19–4.21, P = 0.01) but not OS (Table 3).

**Table 2:** Risk factor determination by univariate analysis for DFS or OS

	DFS			OS		
	P	HR	95%CI	P	HR	95%CI
**Age (y)**						
< 60	1			1		
≥ 60	0.60	1.16	0.66–2.05	0.34	1.38	0.71–2.69
**Gender**						
Male	1			1		
Female	0.01^*^	0.39	0.20–0.76	0.08	0.52	0.25–1.07
**Type of operation**						
Laparotomy	1			1		
Laparoscopy	0.02^*^	0.51	0.28–0.90	0.02^*^	0.46	0.23–0.89
**Tumor location**						
Right	1			1		
Left	0.97	0.99	0.53–1.82	0.59	0.81	0.39–1.71
**History of smoking**						
Current + past	1			1		
Never	0.86	0.96	0.54–1.71	0.58	0.83	0.43–1.60
**History of drinking**						
Current + past	1			1		
Never	0.02^*^	0.51	0.30–0.88	0.15	0.63	0.34–1.19
**Histological grade**						
Well + moderate	1			1		
Poor	0.44	1.35	0.63–2.86	0.56	1.30	0.54–3.10
**Mucinous elements**						
Without	1			1		
With	0.85	1.07	0.52–2.20	0.81	1.11	0.49–2.50
**T stages**						
T_1_ + T_2_	1			1		
T_3_ + T_4_	0.07	2.56	0.92–7.09	0.07	3.79	0.91–15.75
** N stages**						
N_0_	1			1		
N_1+_N_2_	< 0.01^*^	2.55	1.45–4.50	< 0.01^*^	2.87	1.47–5.60
**TNM stages**						
I + II	1			1		
III	< 0.01^*^	2.55	1.45–4.50	< 0.01^*^	2.87	1.47–5.60
**BMI (kg/m** ^**2**^ **)**	0.44	0.97	0.89–1.05	0.15	0.93	0.84–1.03
**IL-6 level**						
Low	1			1		
High	0.02^*^	1.96	1.13–3.38	0.14	1.62	0.86–3.05

^*^with significant difference DFS: disease free survival; OS: overall survival; BMI: body mass index

**Table 3:** Risk factor determination by multivariate analysis for DFS or OS

	DFS			OS		
	P	HR	95%CI	P	HR	95%CI
**Gender**						
Male	1					
Female	< 0.01	0.32	0.16–0.62			
** N stages**						
N_0_	1			1		
N_1+_N_2_	< 0.01^*^	2.72	1.52–4.86	< 0.01^*^	2.70	1.38–5.29
**IL-6 level**						
Low	1					
High	0.04^*^	1.81	1.03–3.15			

^*^with significant difference DFS: disease free survival; OS: overall survival; BMI: body mass index

## Discussion

In this study, we demonstrated that only the postoperative level of IL-6 was a useful prognostic marker in CRC in contrast to CRP and PCT. Patients with a relatively low level of IL-6 had significantly superior survival to those with a high level, and IL-6 was an independent risk factor for DFS. To the best of our knowledge, this is the first report to concurrently compare the prognostic value of CRP, PCT and IL-6 in CRC.

Previously, the prognostic values of CRP, PCT and IL-6 have been individually explored in CRC in preoperative settings; however, only a few studies have been conducted in postoperative settings [22–27]. For example, McSorley et al. conducted a study with 377 stage 0-III patients and found that postoperative CRP > 150 mg/L at 4 d was independently associated with poor DFS [22]; in addition, other studies have also indicated that postoperative CRP could be useful in predicting DFS [23–25] or OS [26]. In addition, Bae et al. performed a study with 248 stage I-IV patients and found that postoperative PCT was a risk factor for OS; however, it was not an independent factor [27]. Notably, no reports to date have explored the role of postoperative IL-6 alone. Interestingly, two reports have concurrently explored these markers (CRP and IL-6), as mentioned previously [16, 17], but with several limitations. One study included stage IV patients [16], who exhibited different survival rates than others [28], and the other study included patients who received additional treatments (such as radiation and chemotherapy) except for surgery [17]. In our study, we included stage I-III patients who underwent surgery alone and concurrently compared the prognostic value of CRP, PCT and IL-6. The results indicated that IL-6 was significant in predicting DFS, which was partially consistent with these reports [16, 17]. Furthermore, we did not identify any significant differences in the collected clinicopathological parameters, including gender, history of smoking, and BMI, among the low or high level IL-6 subgroups, which was also partially consistent with the results of a previous study [17].

It was notable that the prognostic efficacy of CRP and PCT in CRC was inferior to that of IL-6 in the postoperative scenario in our study. In fact, although previous studies have indicated that CRP and PCT were correlated with the outcome either preoperatively [4, 29–31] or postoperatively [23–27] in CRC, there have also been studies suggesting that these factors would be more useful in predicting infective complications [12, 32–34] and may not be associated with disease recurrence [33] or outcome [7, 34–37]. Moreover, one study indicated that IL-6 was the only independent risk factor for survival in contrast to CRP [7]. Additionally, although it was observed that CRP and IL-6 increased within 4 and 24 h, respectively, after the operation [38], CRP normalized within 10 d, but IL-6 did not, which was particularly evident in patients with significantly high preoperative levels [39]. These studies may support the notion that IL-6 would be more robust in predicting survival in CRC to some extent.

Generally, circulating IL-6 levels were found to correlate with systemic inflammatory responses in CRC patients undergoing surgery [40], and it has broad functions in CRC development, such as in promoting tumor growth [41], spreading [42], and angiogenesis [43], as well as in regulating treatment resistance [44, 45]. In recent years, cancer dissemination was found to occur at the very beginning of the disease in CRC [46]. These detached cells, which are also known as circulating tumor cells (CTCs), play a pivotal role in disease recurrence and treatment failure [47, 48]. Interestingly, systemic inflammation can contribute to the metastatic colonization of CTCs [49]. Based on these facts, it is plausible that patients with a surge in IL-6 after surgery are at high risk of cancer metastasis, and these patients can exhibit poor outcomes. Additionally, cancer stem cells (CSCs) were identified as the “true bad seeds” in CRC [50] and are considered to be the source of cancer initiation, recurrence and treatment resistance [51]. Notably, some studies have indicated that a proportion of CTCs are actually CSCs [52, 53], and IL-6 was found to be involved in the expansion [54, 55], promotion [56] and support [57] of these cells. Thus, we speculate that high levels of IL-6 can contribute to the evolution of these CSCs and can also result in poor patient survival. In addition, it was observed that lymphocyte counts as well as T-cell subset counts significantly decline in CRC patients after surgery [38]; additionally, some subsets, such as CD8 + cytotoxic T-lymphocytes, are important in recognizing CSCs [58] and are closely correlated with prognosis [59]. Interestingly, it was also reported that inflammatory conditions not only resulted in a decreased proportion of circulating lymphocytes (although not reported in CRC) [60, 61] but also impaired the anticancer function induced by these cytokines [62]. In our study, the level of IL-6 was observed to be negatively correlated with the ALCs, and although such a correlation seems temporary, it would also partially support its role in survival. Additionally, numerous studies have validated the prognostic value of preoperative inflammation-related markers such as the NLR and LMR in CRC, and it has been generally suggested that a high NLR or a low LMR correlates with poor survival in patients [63–66]. Interestingly, an increasing number of reports have also postoperatively explored the prognostic value of these markers. For example, in a study with 176 stage II cases, Hayama et al. found that postoperative (7 d after surgery) high NLR is a significant independent indicator for shortened relapse-free survival (RFS) [10]; furthermore, in a study with 568 stage III cases, Yasui et al. found that postoperative NLR and LMR were more accurate in predicting RFS or OS in contrast to preoperative factors [9]. In our study, no prognostic value was observed for either NLR or LMR (both preoperatively and postoperatively), which is possibly due to the small sample size, as in previous studies [67–69]. Furthermore, we also demonstrated no correlation of the postoperative IL-6 level with the NLR or LMR (data not shown); thus, its role in prognosis cannot be explained by these markers. We advocate that a larger sample study should be conducted to validate these findings in the future.

Our study has some clinical implications. First, when considering that the bowel is the source of the IL-6 response to surgical trauma in colorectal surgery [70], it is important to select appropriate surgical approaches for these patients. For example, laparoscopic resection is preferred in patients with right-sided lesions due to its advantages in reducing trauma and the systemic inflammatory response [71]. Second, for patients with an obviously high postoperative IL-6 level, it is important to tailor the treatment strategies due to the notorious role of IL-6 in treatment resistance [46, 47] and other functions [41–43]. Third, colorectal adenoma was regarded as a risk factor for CRC [72] and was detected in 7.4–52.5% of patients under colonoscopy [73]; in addition, it was also found that up to 39.4% of CRC patients can develop adenomas in postoperative surveillance [74, 75]. Interestingly, IL-6 was observed to be significantly elevated in patients with colorectal adenoma [76], and a decrease in IL-6 was negatively associated with high-risk and advanced adenoma recurrence in a previous study [77]. Based on these facts, it is important in the clinical setting to screen colorectal adenomas in CRC patients with a high postoperative level of IL-6. There were also some other limitations to our study. First, this study included a limited sample and was retrospectively conducted, and many other factors can bias the results. Second, although a long duration of high postoperative IL-6 levels was observed in CRC patients after surgery, the level of IL-6 normalized at approximately 43 d in most cases [39]. Therefore, it would be more valid to comprehensively analyze the value of these different time points in the clinical setting.

## Conclusion

Overall, our results suggested that in contrast to CRP and PCT, in postoperative settings, only IL-6 was found to be a significant prognostic factor, and patients with a lower IL-6 level could have a good DFS. However, more studies with large samples are still needed to validate our findings in the future.

## Data Availability

The datasets generated or analyzed during the current study are available from the corresponding author on reasonable request.
